# Digitale Versorgungsformen zur Personalisierung der stationsäquivalenten Behandlung

**DOI:** 10.1007/s00115-021-01100-5

**Published:** 2021-03-17

**Authors:** Christian Rauschenberg, Dusan Hirjak, Thomas Ganslandt, Julia C. C. Schulte-Strathaus, Anita Schick, Andreas Meyer-Lindenberg, Ulrich Reininghaus

**Affiliations:** 1 Abteilung Public Mental Health, Zentralinstitut für Seelische Gesundheit, Medizinische Fakultät Mannheim, Universität Heidelberg, J5, 68159 Mannheim, Deutschland; 2 Department of Psychiatry and Neuropsychology, School for Mental Health and Neuroscience, Maastricht University, Maastricht, Niederlande; 3 Klinik für Psychiatrie und Psychotherapie, Zentralinstitut für Seelische Gesundheit, Medizinische Fakultät Mannheim, Universität Heidelberg, Mannheim, Deutschland; 4 Heinrich-Lanz-Zentrum für Personalisierte Medizin, Medizinische Fakultät Mannheim, Universität Heidelberg, Mannheim, Deutschland; 5 ESRC Centre for Society and Mental Health, King’s College London, London, Großbritannien; 6 Centre for Epidemiology and Public Health, Health Service and Population Research Department, Institute of Psychiatry, Psychology & Neuroscience, King’s College London, London, Großbritannien

**Keywords:** eHealth, Hometreatment, Mobile health, Telemedizin, Ecological momentary intervention, eHealth, Digital intervention, Mobile health, Telemedicine, Ecological momentary intervention

## Abstract

**Hintergrund:**

Die stationsäquivalente psychiatrische Behandlung (StäB) wurde 2018 als Krankenhausleistung für Menschen eingeführt, die die Kriterien einer stationären Behandlung erfüllen. Die rasanten Fortschritte im Bereich der Informations- und Kommunikationstechnologie bieten neue Chancen für innovative digitale Versorgungsangebote wie telemedizinische, eHealth- oder mHealth-Verfahren.

**Ziel der Arbeit:**

Diese Übersichtsarbeit soll einen umfassenden Überblick über neue digitale Versorgungsformen geben, die zur Personalisierung der StäB bei schweren psychischen Erkrankungen beitragen und somit klinische und soziale Outcomes verbessern sowie direkte und indirekte Kosten reduzieren könnten.

**Methode:**

Diese Arbeit basiert auf einer selektiven Literaturrecherche (Narratives Review).

**Ergebnisse:**

Es wurden vier primäre digitale Versorgungsformen identifiziert, die in der StäB gewinnbringend genutzt werden könnten: (1) Kommunikation, Behandlungskontinuität und -flexibilität durch Online-Chat und Videotelefonie, (2) Monitoring von Symptomen und Verhaltensweisen in Echtzeit durch Anwendung des ambulatorischen Assessments („ecological momentary assessment“ [EMA]), (3) Nutzung multimodaler EMA-Daten für die Generierung von personalisiertem Feedback über subjektives Erleben und Verhaltensmuster sowie (4) auf Person, Moment und Kontext zugeschnittene, adaptive ambulatorische Interventionen („ecological momentary interventions“ [EMIs]).

**Diskussion:**

Digitale Versorgungsformen haben erhebliches Potenzial die Effektivität und Kosteneffektivität der StäB zu steigern. Ein wichtiger nächster Schritt besteht darin, die Anwendung dieser Versorgungsformen im Bereich der StäB zu modellieren und deren Qualität aus Sicht der Patient*innen, Sicherheit und initiale Prozess- und Ergebnisqualität sowie Implementierungsbedingungen sorgfältig zu untersuchen.

## Abstract (en)

**Background:**

Ward-equivalent treatment (StäB), a form of crisis resolution and home treatment in Germany, has been introduced in 2018 as a new model of mental health service delivery for people with an indication for inpatient care. The rapid progress in the field of information and communication technology offers entirely new opportunities for innovative digital mental health care, such as telemedicine, eHealth, or mHealth interventions.

**Objective:**

This review aims to provide a comprehensive overview of novel digital forms of service delivery that may contribute to a personalized delivery of StäB and improving clinical and social outcomes as well as reducing direct and indirect costs.

**Method:**

This work is based on a narrative review.

**Results:**

Four primary digital forms of service delivery have been identified that can be used for personalized delivery of StäB: (1) communication, continuity of care, and flexibility through online chat and video call; (2) monitoring of symptoms and behavior in real-time through ecological momentary assessment (EMA); (3) use of multimodal EMA data to generate and offer personalized feedback on subjective experience and behavioral patterns as well as (4) adaptive ecological momentary interventions (EMI) tailored to the person, moment, and context in daily life.

**Conclusion:**

New digital forms of service delivery have considerable potential to increase the effectiveness and cost-effectiveness of crisis resolution, home treatment, and assertive outreach. An important next step is to model and initially evaluate these novel digital forms of service delivery in the context of StäB and carefully investigate their quality from the user perspective, safety, feasibility, initial process and outcome quality as well as barriers and facilitators of implementation.

## Hintergrund

Eine zentrale Herausforderung im Bereich der öffentlichen psychischen Gesundheit („public mental health“) bleibt die Versorgung von Menschen mit anhaltentenden und schweren psychischen Erkrankungen, die einen chronisch-rezidivierenden Verlauf nehmen. Besonders bei psychotischen und schweren affektiven Erkrankungen sowie Persönlichkeitsstörungen kommt es häufig zu langandauernden Verläufen und Rezidiven, die mit einer erheblichen Krankheitslast für Betroffene, Angehörige und die Gesellschaft insgesamt, u. a. in Form hoher direkter und indirekter Kosten, verbunden sind. Die Faktoren, die einen chronisch-rezidivierenden Krankheitsverlauf begünstigen können, sind vielfältig und Gegenstand aktueller Forschung. Dabei werden neben anderen Faktoren die fehlende Inanspruchnahme vorhandener Versorgungsangebote und Diskontinuitäten in der Behandlung als wichtige Marker einer ungünstigen Prognose diskutiert.

Vor diesem Hintergrund wurde mit dem Inkrafttreten des Gesetzes zur Weiterentwicklung der Versorgung und Vergütung für psychiatrische und psychosomatische Leistungen (PsychVVG) die stationsäquivalente psychiatrische Behandlung (StäB) nach § 115d, SGB V eingeführt, die eine fachpsychiatrische Behandlung psychisch erkrankter Patient*innen im häuslichen Umfeld umfasst, die die Kriterien einer stationären Behandlung erfüllen. Die StäB bietet die Möglichkeit einer aufsuchenden Behandlung und wird von einem multiprofessionellen Team an 7 Tagen der Woche durchgeführt [18]. Laut Gesetzgeber muss die Behandlung hinsichtlich ihrer angebotenen Inhalte, Komplexität und Intensität einer vollstationären Krankenhausbehandlung entsprechen („Krankenhausbehandlung ohne Bett“) und mindestens einmal täglich ein persönlicher Kontakt mit Patient*innen gewährleistet sein.

Dabei sind gemeindepsychiatrische Versorgungsmodelle, die eine aufsuchende Behandlung von Menschen mit akuten psychischen Krisen außerhalb der stationären Versorgung und innerhalb der Gemeinden im direkten Lebensumfeld vorsehen, nicht neu und auf Versorgungsangebote wie das „assertive community treatment“ von Stein und Test in den USA [37] sowie „Intensive-case-management“- bzw. die „Assertive-outreach“-Teams [25] in UK zurückzuführen. Demnach ist die aufsuchende Behandlung bei Menschen in akuten Krisen bereits seit längerem etablierter Bestandteil der psychiatrischen Versorgung in anderen Ländern, die aber meist in ihrem Umfang und ihrer Intensität von der gesetzlich klar definierten StäB zu unterscheiden sind. So gibt es im britischen National Health Service, je nach Art und Schwere der Symptomatik und individuellen Behandlungsbedarfen, sog. „Crisis-resolution“- und „Home-treatment“-Teams, deren Aufgaben und Zuständigkeiten in nationalen Leitlinien beschrieben sind und unter anderem deren Rolle bei der akuten Krisenbewältigung und dem Erstkontakt hervorheben. Die Effektivität und Kosteneffektivität dieser aufsuchenden Versorgungsangebote sind mittlerweile gut dokumentiert [15].

Auch in Deutschland beinhalten ambulante Versorgungsangebote teilweise bereits aufsuchende Versorgungsangebote im alltäglichen Lebensumfeld von Betroffenen, die auch explizit in der S3-Leitlinie „Psychosoziale Therapien bei schweren psychischen Erkrankungen“ empfohlen werden und dazu beitragen können, bestehende Behandlungslücken zwischen der (teil-)stationären und ambulanten Behandlung zu schließen. Auch wenn die deutschlandweite Versorgungsdichte einer koordinierten, multiprofessionellen aufsuchenden Behandlung weiterhin als gering angesehen werden kann, existiert eine Vielzahl von Initiativen aufsuchender Versorgungsangebote, wie beispielsweise die ambulante psychiatrische Akutbehandlung zu Hause (APAH), die an der Klinik Bamberger Hof in Frankfurt a.M. angeboten wird, oder das 2020 vom Arbeitskreis Psychiatrische Institutsambulanzen (BDK, ACKPA, LIPPs) mit Unterstützung der DGPPN vorgestellte Konzept der ambulant-intensiven Komplexbehandlung (AMBI). Die meisten aufsuchenden Versorgungsangebote können allerdings in ihrem Behandlungsspektrum und ihrer Behandlungsintensität stark variieren, wohingegen die StäB eine gesetzlich klar definierte, komplexe, aufsuchende, an 7 Tagen die Woche stattfindende, zeitlich begrenzte Behandlung durch ein fachärztlich geleitetes, multiprofessionelles Team (mindestens 3 verschiedene Berufsgruppen) im privaten Lebensumfeld der Patient*innen darstellt. Obwohl die derzeitige Umsetzung der StäB – wie die restriktiven gesetzlichen Voraussetzungen einer Behandlungsindikation und strikten Vorgaben der Behandlungsintensität – Gegenstand einer anhaltenden Debatte ist [11], könnte die StäB neben anderen aufsuchenden Behandlungsformen ein weiterer wichtiger Schritt hin zu einer verbesserten psychiatrischen Versorgung sein [17] und zu einer verbesserten Translation der Versorgungsangebote in das häusliche und persönliche Umfeld führen und damit den Einbezug von für Verlauf und Outcome relevante Kontextfaktoren in die Therapieplanung vereinfachen. Durch eine Integration mit stationären, teilstationären, ambulanten und gemeindepsychiatrischen Versorgungsangeboten (wie beispielsweise im Rahmen von Track-Einheiten [13]), könnte insbesondere die StäB auch zu einer erhöhten Behandlungskontinuität beitragen [4].

Ein im Jahre 2019 erschienenes Eckpunktepapier, das von 22 Fachgesellschaften verfasst wurde und sich mit der Umsetzung der StäB in Deutschland befasst, kam zu dem Schluss, dass in vielen Bereichen allerdings noch Handlungsbedarf besteht [8]. Insbesondere die statische Festschreibung der gesetzlich geforderten Behandlungsintensität, der mangelnde Einbezug ambulanter Leistungserbringer und die erschwerten Bedingungen einer nahtlosen Weiterbehandlung zwischen Sektoren bei notwendigen Änderungen des Behandlungssettings wurden hierbei kritisiert und hieraus Prinzipien einer strukturierten sektorenübergreifenden Zusammenarbeit konsentiert. Außerdem stellen das Fehlen an Tools zur mobilen Dokumentation und Teamkommunikation, ein Mangel an Informationen und eine unklare Aufgabenverteilung weitere Barrieren bei der Implementierung der StäB dar [18] – auch wenn einige dieser Hürden seit Publikation des Eckpunktepapiers überwunden sind und manche StäB-Teams bereits mit modernen Kommunikations- und Dokumentationsmitteln arbeiten.

Gleichzeitig haben die rasanten Fortschritte im Bereich der Informations- und Kommunikationstechnologie über die letzten Jahre völlig neue Möglichkeiten der Digitalisierung und Personalisierung der Gesundheitsversorgung geschaffen, die zunehmend an Bedeutung gewinnen [24]. Dabei spielen telemedizinische und internetbasierte Versorgungsangebote (eHealth-Verfahren) sowie Gesundheits- und Medizin-Applikationen (GuMAs; mHealth-Apps) eine besonders wichtige Rolle. Insbesondere GuMAs ermöglichen eine niedrigschwellige, zeitnahe und adaptive Versorgung, die auf die individuellen Bedürfnisse und sozialen Kontexte des alltäglichen Lebens zugeschnitten direkt und schnell angeboten werden können.

Digitale Versorgungsangebote, die mit großem Potenzial für einen Innovationsschub im Rahmen der digitalen Transformation des Gesundheitswesens einhergehen, könnten auch im Kontext der StäB zur fruchtbaren Anwendung kommen und die bislang in Deutschland nur sehr begrenzt untersuchte, aber in internationalen Studien gut dokumentierte Effektivität und Kosteneffektivität aufsuchender Versorgungsangebote steigern. So könnten digitale Versorgungsformen zu einer weiteren Personalisierung der StäB beitragen und somit verbesserte, explizit auf die Bedürfnisse der Patient*innen ausgerichtete Versorgungsangebote schaffen, die in der alltäglichen Lebenswelt verankert sind und in Echtzeit angeboten werden können – auch über den ohnehin schon sehr persönlichen, täglichen Kontakt mit dem multiprofessionellen Team im häuslichen Umfeld hinaus. Somit könnten klinische und soziale Outcomes verbessert sowie direkte und indirekte Kosten reduziert werden. Im Vergleich zu vielen anderen ambulanten Therapieformen eröffnet gerade das bedarfsorientierte und multiprofessionelle Setting aufsuchender Behandlungsformen neue Möglichkeiten, die psychiatrische Behandlung um neue digitale Versorgungsformen zu ergänzen.

### Ziel der Arbeit

Ziel dieser Arbeit ist es, einen umfassenden Überblick über innovative digitale Versorgungsformen zu geben, die zur Steigerung der Effektivität und Kosteneffektivität der StäB in der alltäglichen Lebenswelt in Echtzeit bei Menschen mit schweren und anhaltenden psychischen Erkrankungen beitragen könnten. Diese Arbeit ist aus der Perspektive der mit psychisch kranken Menschen arbeitenden Ärzt*innen, Psycholog*innen und Wissenschaftler*innen entstanden.

## Methodik

Es wurde ein narratives Review durchgeführt. Für diese Arbeit wurde eine selektive Literaturrecherche (Stichtag 30.09.2020) in den Datenbanken MEDLINE und PsycINFO durchgeführt. Hierbei wurden die folgenden drei Suchkonzepte mithilfe von Schlüsselwörtern miteinander kombiniert: Begriffe für (1) psychiatrische Erkrankungen, (2) Telemedizin und eHealth-/mHealth-Interventionen und (3) aufsuchende Behandlungsformen. Die in dieser selektiven Literaturübersicht enthaltenen Artikel wurden nicht auf systematischer Basis ausgewählt, und es wird nicht davon ausgegangen, dass die rezensierte Evidenz erschöpft ist – besonders in Bezug auf den möglichen Nutzens digitaler Versorgungsformen in anderen Behandlungssettings. Die identifizierten Artikel wurden im Konsens zwischen den Autor*innen diskutiert.

## Ergebnisse

### Innovative digitale Versorgungsformen zur Personalisierung der StäB

Die schnell wachsende Zahl von Forschungsarbeiten zu digitalen Technologien in der Versorgung von Menschen mit psychischen Erkrankungen ist bemerkenswert. Im Rahmen unserer selektiven Übersichtsarbeit ließen sich vier primäre digitale Versorgungsformen identifizieren, welche in der Zukunft die therapeutischen Kontakte im Kontext der StäB intensivieren, die Verfügbarkeit der Therapeut*innen steigern, die klinischen und sozialen Outcomes verbessern sowie direkte und indirekte Kosten reduzieren könnten: (1) *Kommunikation, Behandlungskontinuität und -flexibilität *durch Online-Chat und Videotelefonie, (2) *Monitoring* von Symptomen und Verhaltensweisen in Echtzeit durch Anwendung des ambulatorischen Assessments („ecological momentary assessments“ [EMA]), Nutzung multimodaler EMA-Daten für (3) die Generierung von personalisiertem *Feedback* über subjektives Erleben und Verhaltensmuster sowie (4) auf Person, Moment und Kontext zugeschnittene, adaptive *ambulatorische Interventionen *(„ecological momentary interventions“). In den folgenden Abschnitten werden die möglichen Anwendungsbereiche in der StäB vorgestellt und unter Würdigung medizinischer, ethischer, und datenschutzrechtlicher Aspekte diskutiert.

### Kommunikation, Behandlungskontinuität und -flexibilität

Internationale Studien haben die Häufigkeit, Verfügbarkeit und Flexibilität des Kontakts mit einem multiprofessionellen Team sowie die Versorgung nicht nur klinischer, sondern auch sozialer Bedürfnisse als effektive Versorgungskomponenten der aufsuchenden Krisenbehandlung identifiziert, die entscheidend für die Reduktion der Wiederaufnahme- und Therapieabbruchraten sein können [36]. Auch in den ersten Implementierungsstudien zur StäB in Deutschland wurde die erschwerte Kommunikation des multiprofessionellen Teams mit Patient*innen als hinderlich wahrgenommen und Herausforderungen in Bezug auf die räumliche Entfernung zwischen Behandelnden und Patient*innen genannt [3].

Neue digitale Versorgungsformen haben nicht das Ziel, den direkten Kontakt zwischen Patient*innen und Therapeut*innen der StäB zu ersetzen, sondern könnten dabei helfen, die Häufigkeit und v. a. auch die Flexibilität des Kontakts mit dem multiprofessionellen StäB-Team durch Online-Chats und Videotelefonie (beispielsweise unter Verwendung zertifizierter digitaler Gesundheitsanwendungen) zu erhöhen und dadurch sowohl Behandlungskontinuität als auch Kosteneffektivität zu optimieren. Hierbei haben bereits erste Studien gezeigt, dass die Integration telemedizinischer Aspekte in aufsuchende Versorgungsangebote die Effektivität [19] und Kosteneffektivität [31] erhöhen kann. Gerade in ländlichen Regionen oder Städten mit hoher Verkehrsdichte, in denen ein zweiter Besuch am Nachmittag aus zeitlichen und personellen Gründen nicht möglich ist, könnten moderne digitale Technologien dazu beitragen, erneut mit Patient*innen der StäB in (therapeutischen) Kontakt zu treten. Darüber hinaus könnten moderne digitale Technologien zu einer Steigerung der Erreichbarkeit und Verkürzung der Reaktionszeit auf Anliegen der Patient*innen führen. Ferner könnte der mögliche Informationsverlust (wie es aus den Patient*innenübergaben auf Station bekannt ist) zwischen den Mitgliedern des StäB-Teams minimiert werden. Nicht zuletzt könnten diese Kommunikationsdienste auch dazu beitragen, die Sicherheit der StäB für Patient*innen und Behandelnde zu erhöhen, da im Gegensatz zu analogen Kommunikationsformen durch Integration einer digitalen Alarmfunktion die „Hilfe durch Knopfdruck“ möglich wird. Allerdings sind die Qualität aus Sicht der Patient*innen, Sicherheit, Prozess- und Ergebnisqualität sowie Kosteneffektivität eines solchen Ansatzes in der StäB bislang nicht untersucht.

### Digitales Monitoring

Der therapeutische Nutzen und die Kosteneffektivität [15] der aufsuchenden Versorgungsangebote sind mittlerweile gut dokumentiert, könnten jedoch durch den Einsatz des digitalen Monitorings durch Nutzung des ambulatorischen Assessments („ecological momentary assessment“ [EMA]; [40]) weiter gesteigert werden. Weil das therapeutische Team nicht wie auf einer psychiatrischen Station rund um die Uhr das Verhalten und die Symptome der Patient*innen beobachten bzw. erfragen kann, könnten sowohl Patient*innen als auch das StäB-Team von diesen neuen Möglichkeiten profitieren [3].

Ein zentraler Punkt des ambulatorischen Assessments ist, dass das psychische Erleben und Verhalten von Personen als im Kontext situiert verstanden wird [32]. Dabei können Gedanken, Gefühle und Verhalten, einschließlich deren Veränderungen über die Zeit und in Abhängigkeit der alltäglichen Lebenswelt, durch ambulatorische Befragungen im am häufigsten genutzten EMA-Design zu zufälligen Zeitpunkten über einen Zeitraum von mehreren Tagen mithilfe einer Smartphone-App ökologisch valide erfasst werden. Viele der psychopathologischen Symptome und alltagspraktischen Defizite werden nämlich erst im häuslichen Umfeld der Patient*innen für den Therapeuten/die Therapeutin sichtbar. So könnten mithilfe von EMA beispielsweise soziale Ängste, psychotische Symptome, interaktionelle Schwierigkeiten, problematische Verhaltensweisen oder Vermeidungsverhalten in Echtzeit erfasst und an das therapeutische Team rückgemeldet werden. Die Nutzung des Monitorings könnte demnach zu einer umfassenderen Beurteilung der natürlichen Lebenswelt der Patient*innen beitragen, weil sie nicht nur das subjektive Erleben, sondern auch alltägliche Lebensereignisse erfasst, die mit dem subjektiven Erleben der Patient*innen verbunden sind.

Auch die Erhebung passiver Daten, wie die kontinuierliche Erfassung von Aktivitätsmustern und physiologischen Parametern mittels Beschleunigungssensor, Rotationssensor, Pulsmesser oder GPS, gewinnt zunehmend an Bedeutung [20]. Evidenz zur Sicherheit, Akzeptanz und Validität dieser ambulatorischen Erhebungsmethode liegt aus einer Vielzahl von Studien auch bei Patient*innen mit schweren psychischen Erkrankungen vor und ist bisher vor allem zur Erforschung möglicher Mechanismen der Entwicklung und Aufrechterhaltung schwerer psychischer Erkrankungen, wie der emotionalen Stresssensitivität, genutzt worden [29, 33].

Die gewonnenen multimodalen Datensätze ermöglichen ein repräsentatives Bild des Erlebens und Verhaltens der Patient*innen im alltäglichen Leben und könnten im Kontext der StäB entscheidend dazu beitragen, Versorgungsbedarfe frühzeitig zu erkennen und auf Verschlechterungen der Symptomatik und anderer wichtiger Outcomes zeitnah reagieren zu können, gerade wenn – wie bei der StäB – die Behandelnden nicht (immer) unmittelbar vor Ort sind. Darüber hinaus könnten die kontinuierlich erhobenen Daten des ambulatorischen Assessments dazu genutzt werden, dass Ärzt*innen und Psycholog*innen bereits vor der nächsten StäB-Visite Informationen über das subjektive Befinden, Verhalten und die psychopathologischen Symptome vom Vortag einsehen und analysieren können. Auf diesen Informationen basierend können medizinische und kognitiv-verhaltenstherapeutische Interventionen (z. B. Expositionsbehandlung) geplanter, überlegter und zielgerichteter umgesetzt werden. Auch die Identifizierung und Formalisierung kritischer Übergangsphasen („early warning signs“) könnten durch das Monitoring von Symptomen und Verhaltensweisen unterstützt werden und dabei hilfreich sein, eine mögliche Verschlechterung der Symptome automatisiert an das StäB-Team zu melden, um möglichst zeitnah intervenieren zu können. Auch für andere an der StäB beteiligten Berufsgruppen kann das Monitoring hilfreich und ressourcensparend sein. Beispielsweise können Ergotherapeut*innen bei der Durchführung von Funktionsanalysen unterstützt werden, weil Daten aus dem häuslichen Umfeld in Echtzeit verfügbar sind. Ebenfalls könnte die Rückmeldung täglicher Aktivitäten bei der Planung physiotherapeutischer Maßnahmen helfen.

Bisher sind diese neuartigen Möglichkeiten des digitalen Monitorings im Kontext aufsuchender Versorgungsangebote nur sehr begrenzt untersucht, z. B. im Bereich der Gerontopsychiatrie [30]. Im Bereich der StäB bei langandauernden und schweren psychischen Erkrankungen wie z. B. Schizophrenie gibt es bislang keine Evidenz zur Qualität aus Sicht der Patient*innen, Sicherheit sowie Prozess- und Ergebnisqualität. Ergebnisse von Übersichtsarbeiten über die Nutzung des Monitorings in anderen Bereichen der psychiatrischen Versorgung deuten jedoch auf eine hohe Akzeptanz und Effektivität hin, wobei randomisierte kontrollierte Studien sehr selten sind und Langzeiteffekte bisher wenig untersucht wurden [10, 16]. Dabei muss sich das digitale Monitoring nicht ausschließlich auf die Erfassung von psychopathologischen Symptomen und Defiziten im Alltag beschränken, sondern könnte das multiprofessionelle Team auch bei weiteren wichtigen Aufgaben, wie der psychometrischen und neuropsychologischen Diagnostik, Dokumentation von Pflegeprozessen, Beobachtung der Medikamentenadhärenz und Sozialanamnese, unterstützen [5]. Das systematische Monitoring dieser für die Behandlung wichtigen Aspekte würde zu einer Informationsanreicherung führen, die, sinnvoll aufbereitet, vom gesamten multiprofessionellen Team für eine intensivere und alltagsnahe Versorgung genutzt werden könnte.

### Digitales Feedback

Basierend auf den multimodalen EMA-Daten des Monitorings und deren automatisierte Auswertung könnte Patient*innen ein detailliertes digitales Feedback zu ihrem Erleben und Verhalten sowie ihren alltäglichen Aktivitäten und ihrer sozialen Situation per Smartphone-Applikation zur Verfügung gestellt werden. Im Kontext der StäB könnte dies in Form gut aufbereiteter, visualisierter Stimmungs- und Verhaltensprofile geschehen, die auch in Kombination mit herkömmlichen Versorgungsangeboten – wie beispielsweise im Rahmen der Psychoedukation oder kognitiv-behavioralen Ansätzen – durch Kliniker*innen sinnvoll genutzt werden könnten [16]. Das gemeinsame Besprechen des personalisierten Feedbacks könnte Patient*innen Einblicke in und Reflexion über ihr subjektives Erleben und ihre eigenen Verhaltensmuster ermöglichen [6], die nicht durch selektive oder andere kognitive Verzerrungen („cognitive biases“) beeinflusst werden. Dabei könnte sich das Feedback auf einen einfachen Wochenüberblick der berichteten Aktivitäten, Gefühle und Verhaltensweisen beschränken [16]. Auch die visuelle, leicht verständliche Aufbereitung der Veränderung von Gefühlen in Abhängigkeit von Aktivitäten und Kontexten im alltäglichen Leben könnte dazu führen, dass Patient*innen Einsicht in eigene Verhaltenskontingenzen bekommen. Dies könnte auch für Behandelnde eine sinnvolle Ergänzung des Behandlungsspektrums darstellen und dazu beitragen, dass für Patient*innen wichtige Verhaltensänderungen und -auffälligkeiten besser nachvollziehbar sind [39]. Auf solchen EMA-Daten basierend könnte somit auch der Transfer des in den psychotherapeutischen Sitzungen Erlernten in den Alltag besser gelingen. Die Nutzung verschiedenster Formen personalisierten Feedbacks ist in einer Vielzahl von Studien bereits untersucht worden und vielversprechende Ergebnisse zur Akzeptanz und Effektivität liegen vor [16]. Im Bereich der aufsuchenden Behandlung ist allerdings, nach bestem Wissen, bisher keine Studie veröffentlicht worden.

### Ambulatorische Intervention

Digitale Versorgungsformen umfassen nicht nur die Möglichkeit, Symptome und Bedürfnisse in Echtzeit zu erfassen und darauf aufbauend personalisiertes Feedback für Betroffene abzuleiten, sondern auch digitale Interventionen im alltäglichen Leben anzubieten. Dabei sind insbesondere auf Person, Moment und Kontext zugeschnittene, adaptive ambulatorische Interventionen („ecological momentary interventions“ [EMIs] oder neuerdings synonym „just-in-time adaptive interventions“) geeignet [23, 24, 32], digitale Versorgungsformen mit in Echtzeit generierten Daten des ambulatorischen Assessments zu verknüpfen und in Form einer mHealth-App niederschwellig anzubieten [32]. EMIs können als Erweiterung des ambulatorischen Assessments verstanden werden und ermöglichen Interventionen, die adaptiv auf die Bedürfnisse des Alltags ausgerichtet sind und evidenzbasierte Interventionskomponenten etablierter Behandlungsverfahren ins alltägliche Leben der Patient*innen übersetzen.

Dabei gehen EMIs davon aus, dass das psychische Erleben und Verhalten von Personen nicht nur im Kontext der alltäglichen Lebenswelt situiert, sondern auch genau in diesem Kontext am besten veränderbar ist [32]. Aus diesem Grund sind bei EMIs die digitalen Versorgungsangebote der mHealth-App nicht nur jederzeit durch das Smartphone abrufbar, sondern werden besonders auch dann adaptiv angeboten, wenn auf Grundlage von EMA-Daten bestimmte Symptome und Bedürfnisse vorhanden (z. B. Momente stärkerer Symptomatik) oder passive Sensordaten auffällig sind (z. B. geringe körperliche Aktivität über mehrere Stunden am Tag). Dabei kann die Anwendung von Methoden der künstlichen Intelligenz und des maschinellen Lernens dabei helfen, diese adaptiven, auf Person, Moment und Kontext zugeschnittenen EMIs auf intraindividueller Ebene weiter zu personalisieren [27].

Erste Ergebnisse zu EMIs wie ACT-DL („acceptance and commitment therapy in daily life“; [34]) und EMIcompass („ecological momentary intervention on self-compassion“; [26]) sind vielversprechend und zeigen, dass therapeutische Prinzipien und Inhalte erfolgreich ins alltägliche Leben übersetzt werden können und eine hohe Machbarkeit, Akzeptanz und initiale therapeutische Effekte aufweisen. Eine weitere EMI, die die Steigerung des Selbstwertgefühls junger Menschen mit kindlicher Traumatisierung zum Ziel hat, wird derzeit – wie auch ACT-DL und EMIcompass – in einer randomisierten kontrollierten Studie untersucht [7]. Ambulatorische Interventionen im Bereich des Hometreatments sind hingegen selten. Nur vereinzelt wurde berichtet, dass die Anwendung digitaler Interventionen in der aufsuchenden Behandlung im Bereich der Gerontopsychiatrie vielversprechend sein könnte [38]. Allerdings wurden auch hier Implementierungshürden ambulatorischer Intervention beschrieben, die bereits in einem frühen Stadium der Umsetzung der digitalen StäB erkannt und überwunden werden müssen.

Nicht zuletzt könnten Patient*innen auch über eine App auf dem Smartphone an eine mögliche Medikamenteneinnahme, Abgabefristen oder Termine erinnert werden. Zusammengefasst können die beschriebenen digitalen Versorgungsformen dazu beitragen, sowohl die Behandlungskontinuität und therapeutische Wirksamkeit als auch die Kosteneffektivität der StäB zu steigern.

## Diskussion

Im Rahmen unserer selektiven Übersichtsarbeit ließen sich vier primäre digitale Versorgungsformen identifizieren, welche die therapeutischen Kontakte im Kontext der StäB intensivieren, die Verfügbarkeit der Therapeut*innen steigern, die klinischen und sozialen Outcomes verbessern sowie direkte und indirekte Kosten reduzieren könnten. Dabei könnten die digitalen Versorgungsformen zu einer umfassenderen Beurteilung der natürlichen Lebenswelt der Patient*innen beitragen, weil sie nicht nur das subjektive Erleben, sondern auch alltägliche Lebensereignisse in Echtzeit erfassen, die mit dem subjektiven Erleben der Patient*innen verbunden sind. Dabei könnte der ohnehin schon sehr persönliche, tägliche Kontakt mit dem multiprofessionellen Team der StäB im häuslichen Umfeld unterstützt werden und einzelne Komponenten der Behandlung noch besser an Person und Kontext angepasst werden.

Es existieren allerdings einige (überwindbare) Hürden in der Implementierung, die sorgfältig untersucht werden müssen. Erstens, obwohl die StäB den Erhalt der etablierten Lebenswelt der Patient*innen fördert, müssen stets die besonderen Lebensumstände berücksichtigt werden. Viele Patient*innen lassen sich lieber zu Hause als in der Klinik behandeln, weil sie sich außerhalb der Therapiezeiten um ihre minderjährigen Kinder, pflegebedürftigen Angehörige oder Haustiere kümmern müssen. Die Nutzung der identifizierten digitalen Versorgungsformen außerhalb der vereinbarten Therapiezeiten kann zur Überforderung der Patient*innen führen. Darüber hinaus ist die Therapieadhärenz der Patient*innen außerhalb von Studienprotokollen reduziert [22]. Entscheidend ist dabei, dass Betroffene unmittelbar von den beschriebenen digitalen Versorgungsformen profitieren.

Zweitens, es ist wichtig zu beachten, dass diese neuen digitalen Versorgungsformen im Kontext der StäB nicht für jeden Patienten/jede Patientin geeignet sind. Patient*innen der StäB weisen häufig schwer ausgeprägte psychopathologische Symptome (z. B. Angst, Misstrauen, technischer Beeinflussungs- oder Beeinträchtigungswahn [12]), kognitive Beeinträchtigungen (z. B. Schizophrenie, ADHS oder Demenz) und störende Verhaltensweisen (z. B. motorische Tics beim Tourette-Syndrom, Vermeidungsverhalten bei Persönlichkeitsstörungen) auf, die eine erfolgreiche Nutzung dieser Technologien erschweren könnten – auch wenn Studien die Machbarkeit und hohe Akzeptanz digitaler Interventionen bei Menschen mit schweren psychischen Erkrankungen zeigen konnten [24]. Auch die wahrgenommene Kompetenz im Umgang mit Technologien und negative Einstellungen und Überzeugungen zu digitalen Interventionen wurden als wichtige Implementierungshürden beschrieben und könnten durch partizipatorische Elemente bereits während des Entwicklungsprozesses der digitalen Versorgungsformen frühzeitig erkannt und aufgegriffen werden [2]. Drittens, auch die StäB-Mitarbeiter*innen könnten skeptisch sein, weil nicht alle mit moderner Technik umgehen und die erhobenen Daten für sich direkt nutzen können. Hierfür sind regelmäßige Schulungen des multiprofessionellen Teams notwendig, in denen der mögliche Nutzen und konkrete Anwendungsbeispiele im klinischen Alltag besprochen werden.

Viertens, bisher fehlen Daten zu Langzeiteffekten der StäB und einigen der identifizierten digitalen Versorgungsformen. Belastbare wissenschaftliche Evidenz zur Kombination von StäB und digitalen Versorgungsformen würde sicherlich die Akzeptanz bei Patient*innen, Mitarbeiter*innen und den Kostenträgern steigern. Fünftens, ein weiterer wichtiger Faktor des flächendeckenden Angebots der StäB und Integration in das medizinische Versorgungssystem ist die systematische Untersuchung der Kosteneffektivität. Belastbare Kosten-Nutzen-Analysen zu mHealth-Versorgungssystemen fehlen bisher. Lediglich in einer Studie von Simons et al. [35] wurde die Kosteneffektivität des Monitorings und Feedbacks als additive Intervention bei Patient*innen mit Depression untersucht. Sechstens, das Sammeln, Speichern und Verarbeiten von Informationen zur psychischen Gesundheit der Patient*innen ist ein zentrales Thema der Digitalisierung des Gesundheitswesens und sollte durch Beratung durch Fachleute im Bereich der Datensicherheit und des Datenschutzes bereits zu einem frühen Zeitpunkt des Entwicklungsprozesses digitaler Versorgungsformen berücksichtigt werden, um nicht nur die Daten des ambulatorischen Assessments zu sichern und die Einhaltung aller Aspekte der europäischen Datenschutz-Grundverordnung (EU-DSGVO) bzw. der Europäischen Verordnung für Medizinprodukte (Medical Device Regulation) zu gewährleisten, sondern auch das Vertrauen der Patient*innen und des multiprofessionellen Teams in digitale Technologien zu steigern [14].

Aktuell finden digitale Versorgungsangebote bei Menschen mit schwerer und langandauernder psychischer Erkrankung sehr wenig Anwendung in der psychiatrischen Regelversorgung. Dies scheint nicht ausschließlich an krankheitsbedingten Faktoren wie Symptomschwere oder Dauer der Erkrankung zu liegen, sondern könnte auch durch Hindernisse bei der Implementierung in die Regelversorgung (z. B. Datensicherheit, Interoperabilität) und gesetzliche Rahmenbedingungen wie das Medizinproduktgesetz erklärt werden. Vereinzelt werden jedoch telemedizinische Ansätze wie Videokonferenzsoftware bereits genutzt, um die Kommunikation zwischen klinischem Personal und Patient*innen zu unterstützen – wie sich nicht zuletzt seit Ausbruch der COVID-19-Pandemie eindrucksvoll gezeigt hat [28]. Gesundheits- und Medizin-Apps kommen hingegen noch nicht zur routinemäßigen Anwendung. Dies ist besonders deshalb bemerkenswert, weil diese Apps ein zentraler Aspekt des kürzlich in Kraft getretenen Digitale-Versorgungs-Gesetz sind und vielversprechende Ergebnisse zu ihrem möglichen Nutzen vorliegen [41]. Dabei weisen Studien konsistent darauf hin [9], dass digitale Interventionen besonders dann effektiv sind und von Patient*innen akzeptiert werden, wenn sie durch Gesundheitsberufe beratend oder therapeutisch begleitet werden („Blended-care“-Ansatz). Auch bei Menschen mit schwerer und langandauernder psychischer Erkrankung zeigen Übersichtsarbeiten, dass computerbasierte und mobile Intervention von einer Mehrzahl der Patient*innen akzeptiert werden und mit vielversprechenden Outcomes, wie einer Verbesserung positiv psychotischer und depressiver Symptomatik, geringe Krankenhauseinweisungen und einer erhöhten Medikamentenadhärenz, assoziiert sind [1, 21].

Die StäB am Zentralinstitut für Seelische Gesundheit (ZI) versteht sich als eine Erweiterung des Track-Konzeptes [13] und ist eine wertvolle und an die individuellen Bedürfnisse der Patient*innen angepasste Alternative zum klassischen stationären Aufenthalt. Ein stationärer Aufenthalt in einer psychiatrischen Klinik kann gerade für Patient*innen eine Belastung sein, weil sie aus ihrem gewohnten Umfeld herausgenommen und mit anderen fremden und häufig schwer kranken Menschen konfrontiert werden. In der StäB bleibt der Patient/die Patientin im vertrauten Umfeld und kann gezielt an alltagspraktischen Defiziten arbeiten. Gerade sozioökonomisch benachteiligte Patient*innen mit Schizophrenie oder Patient*innen mit somatischen Komorbiditäten können gemeinsam mit Therapeut*innen den Wochenplan für die darauffolgenden Tage besser und kostengünstiger planen. Diese und andere Aspekte könnten mithilfe der identifizierten digitalen Versorgungsformen sinnvoll begleitet werden. Das in diesem Jahr gestartete Projekt „DiSERVE@home“, welches am ZI durchgeführt wird, hat im Lichte dieser Erkenntnisse das übergeordnete Ziel, die Anwendung der in der vorliegenden Übersichtsarbeit identifizierten digitalen Versorgungsformen im Bereich der StäB zu modellieren und deren Qualität aus Nutzersicht, Sicherheit und initiale Prozess- und Ergebnisqualität sowie Implementierungsbedingungen sorgfältig zu untersuchen.

Zusammenfassend weisen die digitalen Versorgungsformen in den hier ausgewählten Anwendungsszenarien für einige Patient*innen Potenzial auf, als besonders wirksam erachtete Versorgungskomponenten der StäB zu verstetigen sowie die Effektivität zu steigern. Außerdem könnte hierdurch eine nachhaltige und skalierbare Implementierung der StäB erleichtert und einer besseren Translation fachpsychiatrischer und psychotherapeutischer Versorgung in die alltägliche Lebenswelt Rechnung getragen werden. Die Anwendung digitaler Versorgungsformen könnte auch zu einer erhöhten wahrgenommenen Selbstständigkeit und einem Empowerment der Patient*innen der StäB führen, da Betroffene die Möglichkeit bekommen, noch aktiver an ihrem eigenen Behandlungsprozess mitwirken zu können. Obwohl dies erst Gegenstand zukünftiger Untersuchungen zur digitalen StäB sein muss, könnte man davon ausgehen, dass die Integration digitaler Versorgungsformen mit geringeren Therapieabbruch- und Wiederaufnahmeraten auf einer psychiatrischen Akutstation und einer erhöhten Behandlungs- und Medikamentenadhärenz assoziiert ist.

## Fazit für die Praxis

- Fortschritte im Bereich der Informations- und Kommunikationstechnologie könnten zur Personalisierung und Verbesserung der Effektivität und Kosteneffektivität der stationsäquivalenten psychiatrischen Behandlung (StäB) beitragen.
- Insbesondere vier digitale Versorgungsformen könnten vom multiprofessionellen Team genutzt werden: (1) Online-Chat und Videotelefonie für eine verbesserte *Kommunikation und Erhöhung der Behandlungskontinuität und -flexibilität*; (2) digitales *Monitoring* von Symptomen und Verhaltensweisen in Echtzeit durch Anwendung des ambulatorischen Assessments, (3) Nutzung dieser multimodalen Daten für die Generierung von personalisiertem *Feedback* über subjektives Erleben und Verhaltensmuster sowie (4) auf Person, Moment und Kontext zugeschnittene, adaptive *ambulatorische Interventionen *(„ecological momentary interventions“ [EMIs]).
- Digitale Versorgungsformen haben auch das Potenzial, als besonders wirksam erachtete Versorgungskomponenten der StäB zu verstetigen und damit eine nachhaltige und skalierbare Implementierung zu ermöglichen.
- Die vielseitigen Möglichkeiten der identifizierten digitalen Versorgungsformen könnten auch im Kontext anderer Behandlungssettings – nicht nur der StäB – ausgelotet und möglicherweise gewinnbringend genutzt werden.
- Ein wichtiger Schritt besteht nun darin, die Qualität aus Sicht der Patient*innen, Sicherheit und initiale Prozess- und Ergebnisqualität dieser digitalen Versorgungsformen sowie Implementierungsbedingungen zu untersuchen.
